# An Unusual Complication of Congenital Diaphragmatic Hernia

**DOI:** 10.1055/s-0037-1607353

**Published:** 2017-10-27

**Authors:** E Tian Tan, Keren Sloan, Kokila Lakhoo

**Affiliations:** 1 Department of Paediatric Surgery, Oxford University Hospitals NHS Foundation Trust, Oxford, Oxfordshire, United Kingdom of Great Britain and Northern Ireland; 2 Department of Paediatric Surgery, University of Oxford, Oxford, United Kingdom of Great Britain and Northern Ireland

**Keywords:** congenital diaphragmatic hernia, complications, gastrointestinal, obstruction, neonatal, surgery

## Abstract

A term newborn was referred to our unit with a postnatal diagnosis of a right-sided congenital diaphragmatic hernia (CDH). She was managed with high-frequency oscillatory ventilation, inotropic support, and nitric oxide, with planned surgical repair when she was medically optimized. On day 6 of life, there was an acute deterioration causing difficulty maintaining adequate ventilation and the infant requiring increasing analgesia and paralysis, especially during abdominal examination. A repeat X-ray showed distended bowel loops in the right hemithorax when compared with previous films raising suspicion of bowel obstruction. The infant proceeded to emergency laparotomy in the neonatal intensive care unit. She was found to have a right-sided Bochdalek (posterolateral) defect. The entire small bowel was within the thoracic cavity and appeared dusky secondary to obstruction caused by compression of a herniated right liver lobe against the hernia defect. Bowel perfusion improved after reduction and a BioDesign patch was used to repair the defect. The infant went on to have a straightforward recovery and was transferred to her local hospital for ongoing care on day 17. Bowel obstruction is an uncommon complication in the perinatal period in infants with CDH. A high index of suspicion for bowel compromise is needed in neonates who deteriorate acutely after a period of stabilization. Imaging should be obtained as soon as possible and early surgical intervention may be life-saving.

## Introduction

Congenital diaphragmatic hernia (CDH) is a congenital anomaly that occurs in approximately 1 in 2,000 to 4,000 live births. 1 A defect in the diaphragm leads to herniation of abdominal contents into the thorax and affects cardiorespiratory function, primarily in the form of pulmonary hypoplasia and pulmonary hypertension. 2 Gastrointestinal (GI) complications in the perinatal period are not common and only a handful of cases have been reported. 2 3 4 5 6 7 8 We present a case where an infant with postnatal diagnosis of right-sided CDH, who was being optimized medically, subsequently developed acute bowel obstruction as a complication of CDH and required emergency laparotomy.

## Case Report

**Fig. 1:**
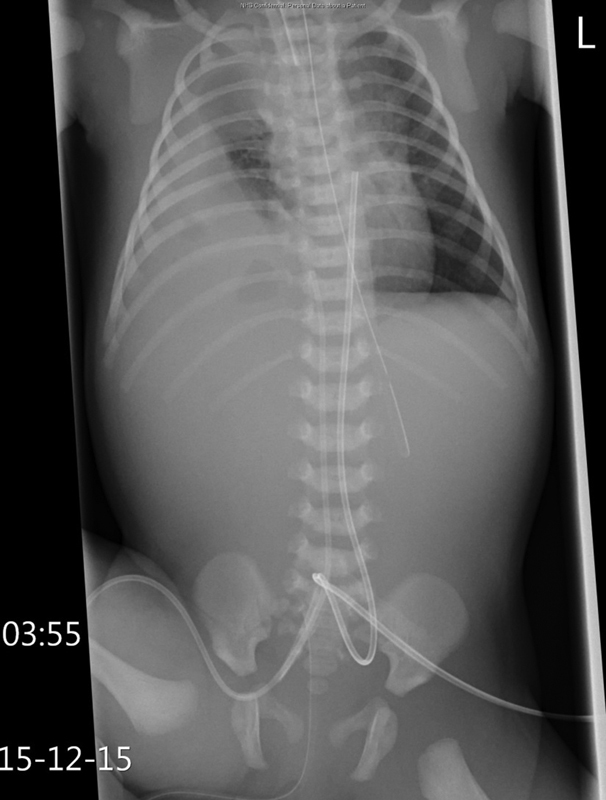
X-ray at birth demonstrating a right-sided CDH. CDH, congenital diaphragmatic hernia.

**Fig. 2:**
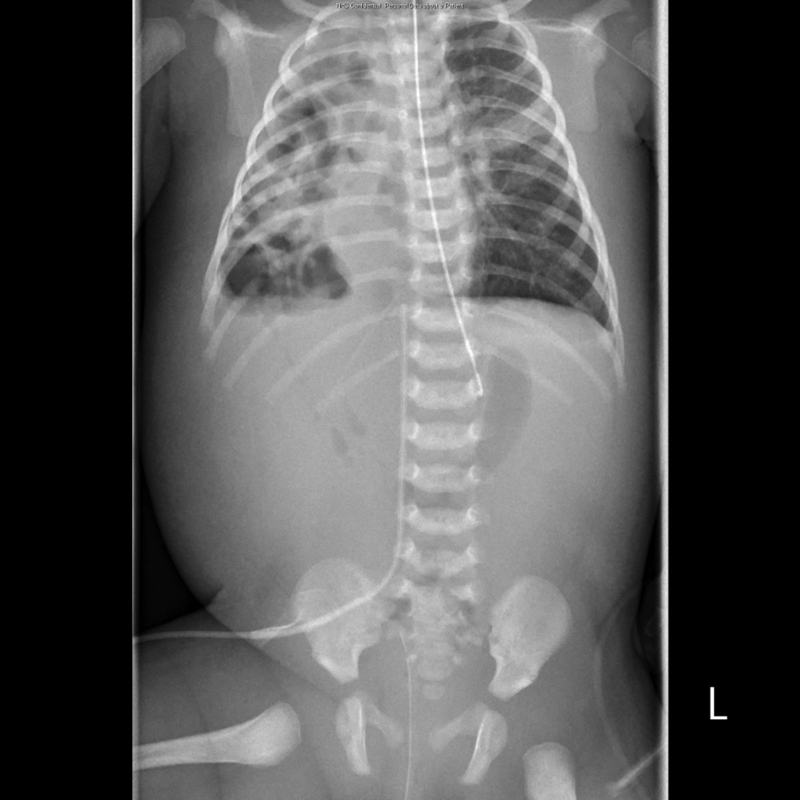
X-ray showing distended bowel loops in right hemithorax prior to emergency surgery in NICU. NICU, neonatal intensive care unit.

A 3.2-kg female infant with normal antenatal scans was born at 41 + 2 weeks to a gravida 2, para 1 woman via spontaneous vaginal delivery in a midwife-led unit. At birth, she was noted to have increased work of breathing and saturation of 32% on room air. Apgar score was 7 at 1 and 5 minutes, respectively. A chest X-ray showed a right-sided CDH ( Fig. 1 ). She was transferred to our unit for further management. On arrival to neonatal intensive care unit (NICU), she was ventilated via high-frequency oscillatory ventilation (HFOV) with FiO _2_ (fraction of inspired oxygen) 100%, mean airway pressure 18, delta-P 30, rate 10 Hz, and iNO (inhaled nitric oxide) 20 ppm. Dopamine and dobutamine infusion was started for inotropic support. A nasogastric (NG) tube was inserted, and she was started on parenteral nutrition. Echocardiogram showed a structurally normal heart with severe suprasystemic pulmonary pressure (estimated pulmonary arterial pressure, 88 mm Hg) and right ventricular failure. Over the next 48 hours, various inotropes (noradrenaline, milrinone, and hydrocortisone) were added to maintain the blood pressure (BP). A gradual improvement was noted thereafter and pre- and postductal saturations of 92 to 93% were maintained, while FiO _2_ was reduced to 60 to 70% on HFOV. There was good urine output and minimal aspirates from NG tube. On day 6 of life, FiO _2_ had to be increased to 80% to maintain saturations. She was also breaking through sedation, and an increase in heart rate and BP was observed during palpation of the abdomen. The abdomen was more distended and showed signs of guarding. Minimal aspirates were obtained from the NG tube and bowels last opened on day 4. Arterial blood gas reported a pH 7.30, pO _2_ (partial pressure of oxygen) 8.4 kPa, pCO _2_ (partial pressure of carbon dioxide) 8.68 kPa, lactate 1.6, HCO ^3-^ 26.8, and base excess +5.3. Laboratory counts showed hemoglobin (Hb) 154, white blood cell (WBC) 15.3, platelets (Plts) 414, and C-reactive protein (CRP) 0.8. A repeat X-ray ( Fig. 2 ) showed new distended bowel loops in the right hemithorax.

Due to suspicion of acute bowel obstruction, an emergency laparotomy was performed in NICU. A right-sided Bochdalek (posterolateral) defect was found intraoperatively with no sac and no posterior rim. The entire small bowel was within the thoracic cavity and appeared dusky secondary to obstruction caused by compression of a herniated right liver lobe against the hernia defect. Bowel perfusion improved after reduction and application of warm packs. The right lung was estimated to be hypoplastic at 20% using rib spaces. No bowel resection was needed and a biologic hernia graft (BioDesign patch) was used to repair the defect. She went on to have an uncomplicated recovery and was extubated on day 7 post operation, achieved full feeds on day 10, and transferred back to her local hospital for ongoing care on day 17. Postoperative chest X-ray showed a right lung that did not fully reexpand with progressive opacification of the surrounding space in the right hemithorax. This was managed conservatively due to improving respiratory function. She was discharged on high-flow therapy with small amounts of supplemental oxygen.

## Discussion and Conclusion

Bowel obstruction, as a complication of CDH in the perinatal period, is quite unusual. It is more commonly reported as one of the presenting symptoms leading to diagnosis in late-presenting CDH 1 9 10 or as a postoperative complication following surgical repair of CDH. 1 11 Late-presenting CDH is now recognized as a separate clinical entity and presents primarily as chronic respiratory symptoms (frequent chest infections, cough) and/or acute/chronic GI symptoms (bowel obstruction or perforations, regurgitations, abdominal pain) owing to a smaller defect and thus sparing the cardiorespiratory compromise that presents acutely in early life. 1 9 10 A left-sided CDH is more common and is associated with more GI complications due to the protective effect of the liver on the right preventing herniation. 10 Chang et al also postulated that the requirement for fasting in the neonatal CDH group presenting with respiratory distress made it less likely for GI complications to develop. 9

Of the cases that reported bowel or gastric obstruction as a perinatal complication, three presented with symptoms of obstruction (bilious vomiting, lethargy) at early life that led to the diagnosis of CDH. 2 6 8 Three cases were of bowel obstruction or perforation occurring in utero and presenting prenatally 3 4 or immediately at birth. 5 In these cases, surgery was delayed until medically optimized, as there was no immediate threat. All of the above were left-sided CDH with small defects reparable by primary closure and no significant pulmonary hypoplasia. In a case series from a single center, the authors reported five cases of acute intestinal obstruction (involving various parts of the bowel and stomach) in neonates with CDH and concluded that antenatal fetal distress or postnatal instability despite attempts at optimizing cardiorespiratory function should raise suspicion of bowel obstruction. 7

Our case differs from the above case reports, as the bowel obstruction developed acutely in an infant who was otherwise stabilized from a cardiorespiratory functional point of view. There was an absence of bilious vomiting. The primary clue that raised suspicion was of an unsettled neonate, especially during abdominal examination despite being sedated and paralyzed. The mechanism of the obstruction was also quite unusual with the liver being the main culprit possibly due to the larger defect. In conclusion, an infant who acutely deteriorates after a period of stabilization should raise suspicion, and imaging should be obtained as soon as possible. If bowel obstruction was suspected, early surgical intervention can be life-saving.
